# Clinical Observations upon Anæsthesia

**Published:** 1849-01

**Authors:** 


*Clinical Observations upon Anæsthesia.* By Dr. BEAU.—The object of this paper is to exhibit the fact that in certain forms of disease a condition of anæsthesia is present, which has hitherto for the most part escaped attention.

1. *Saturnine Intoxication.* Writers treating upon the deleterious influence exerted by lead upon the system, have noted anæsthesia as an occasional and rare occurrence in individuals who have been long exposed to its operation; and M. Tanquerel states that of 2160 persons suffering under various effects of lead disease, only eleven offered symptoms of deficiency of general insensibility. Nevertheless, anæsthesia is an habitual, if not an essential symptom of impregnation of the system by this metal. M. Beau's attention was at first accidentally directed to the subject, on observing an insensibility to feeling and to pain in other parts of his body. Trying the experiments upon other individuals similarly circumstanced, he found the same result,—pricking, pinching, &c. the surface exciting no sense of pain; and he has now investigated the fact in thirty cases, some being very slight examples of colic, &c. and others of very short duration.

In these cases there are, however, *two varieties* of anæsthesia, viz. insensibility to touch, and insensibility to pain, better termed *analgesia.* The first of these is by far the least common, having been met with in only four of the cases. It is, too, usually partial, extending only over a small portion of the surface; but it announces a far more serious lesion than analgesia. This last is *constantly* found in persons suffering from lead poisoning in any of its degrees. We must, however, not content ourselves with asking the patient whether he feels, but confine our question to the sensation of *pain.* Parts which are thus insensible to pain are so also to *tickling.* This form of anæsthesia may affect the entire surface, being however, most remarkable in the extremities, and especially the upper ones. It may extend even to the mucous membranes, and especially those which are normally endowed with great sensibility—as the uvula, isthmus faucium, nares, or conjunctiva—any of which parts may be tickled without the usual consequences, the patient being still quite conscious of the mere contact. The anæsthesia disappears as the cachectic colour is removed, and the appetite restored; with a slowness proportionate to the age of the patient, or to the severity of his disease. It has disappeared as soon as the sixth day after treatment, and at other times not until the twelfth or fifteenth. With this anæsthesia to excited pain, violent pain of a spontaneous character, as colics, &c. may exist.

2. *Hysteria.* M. Gendrin, in 1846, first stated anæsthesia to be a constant symptom in this disease ; but he did not distinguish between insensibility to pain and insensibility to touch, the latter being very rare, and only observed in very bad cases.

3. *Hypochondriasis.* Anæsthesia is to be observed also in this disease when well marked and of long standing. Besides the diseases mentioned, it will probably be found in *scorbutus* and *pellagra* (both of which not infrequently terminate in paralysis,) and in various other forms of colic besides that from lead. The *nervous delirium* of Dupuytren following traumatic affections is attended with this anæsthesia, and the insensibility to surgical operations manifested by some of the insane may be similarly explained ; and these views throw some light upon the ease with which religious fanatics have at different periods apparently borne the most horrible sufferings.

The above pathological considerations lead to the physiological separation of the sense of touch and of pain ; and this is susceptible of proof by experiment, for after a blow, cut, &c. the pain is distinctly posterior to the perception of the injuring body. "The sense of pain may, so to speak, be considered as annexed to that of touch, and thus is the first to disappear when the innervation does not possess its normal intensity. If the innervation is subjected to still farther diminution, then the sense of touch disappears in its turn, and the insensibility is complete. The object of the sensation of touch is to inform us of the presence of bodies in general, while that of pain has the no less important office of advertising us of the contact of disorganizing bodies." We may then say that there is a paralysis of the sense of pain, just as of the sense of touch, of the special senses, or of the motor powers.—*Archives Gén.*

Since publishing the above communication, M. Beau has found that *typhoid fever* may be added to the diseases accompanied with anæsthesia, which persists even during convalescence.

He has also remarked that *analgesia* in the various cases is more observable in the erect than in the recumbent posture, after exertion, e. g. mounting stairs, and indeed after any action, whether physical or mental, which produces a temporary diminution of strength.—*Brit. and For. Med. Chir. Rev. from Gaz. des Hôp.*

